# Health sector fragmentation: three examples from Sierra Leone

**DOI:** 10.1186/s12992-018-0447-5

**Published:** 2019-01-22

**Authors:** Arwen Barr, Lauryn Garrett, Robert Marten, Sowmya Kadandale

**Affiliations:** 1 Simon Fraser University, 8888 University Drive, Burnaby, Canada; 2 London School of Hygiene and Tropical Medicine, London, England; 3 UNICEF Indonesia, Jakarta, Indonesia

**Keywords:** Health sector fragmentation, External development assistance, Health system strengthening, Sierra Leone

## Abstract

**Background:**

Fragmentation across governance structures, funding, and external actor engagement in Sierra Leone continues to challenge the efficiency and coherence of health sector activities and impedes sustained health system strengthening. Three examples are discussed to highlight the extent, causes, and impacts of health sector fragmentation in Sierra Leone: the community health worker programme, national medical supply chain, and service level agreements.

**Results:**

In these examples we discuss factors contributing to fragmentation, the impact on efficiency of systems and sustainability of interventions, and persistent barriers to achieving sustainable improvements in health system performance. Prolonged external dependence and a proliferation of partner and donor involvement tending towards vertical programming and funding have contributed to this fragmentation.

**Conclusion:**

Alignment of policy and planning initiatives, investment in proactive (to reduce need for reactive) policy and plan development, strengthened partnerships, and strengthened governance and accountability mechanisms offer opportunities for greater health sector integration.

## Key messages

- Health sector fragmentation continues to impede health system strengthening in Sierra Leone.
- Prolonged external dependence and rapid and uncoordinated policy and plan development contribute to distortion, dilution, and diversion of health sector financing, resources, and capacity.
- The Government of Sierra Leone has introduced efforts to strengthen the coordination, integration, and governance of health sector activities and partner engagement but existing fragmentation constrains this progress.
- Alignment of policy and planning initiatives, investment in proactive (to reduce need for reactive) policy and plan development, strengthened partnerships, and accountability mechanisms offer opportunities for greater health sector integration.

## Introduction

Health sector fragmentation across structures, funding, and external actor engagement has been widely discussed in global health literature, often from a high-level perspective [5, 32, 35, 43–46].

In several low-income countries including Sierra Leone, prolonged external dependence, donors’ misalignment, and a proliferation of partner and donor involvement (with tendencies towards vertical programmes) undermine efforts to strengthen health systems [5, 12, 14, 22]. International literature shows that health sector fragmentation contributes to duplication of services, dilution and distortion of limited human and financial resources, and weak coordination between levels of care [35] and is both a product and perpetuator of short-term, siloed, vertical approaches to development [1, 5, 44–46].

The aim of this article is to contribute to the literature on health sector fragmentation a description and analysis of fragmentation in Sierra Leone’s health sector through the review of three examples. The authors have selected three examples to highlight fragmentation throughout Sierra Leone’s health sector, from policy and planning, health system structures, and governance of partner engagement. In these examples we discuss factors contributing to fragmentation, the impact on efficiency of systems and sustainability of interventions, and persistent barriers to achieving sustainable improvements in health system performance. Through this analysis, we identify opportunities to improve health system capacity to not only respond to the next health emergency, but to better protect and respond to the health needs of Sierra Leoneans. Other factors contributing to health sector fragmentation such as Sierra Leone’s colonial history, the civil war, poor infrastructure, shortage of human resources for health, and the current political climate are not explored within this article.

A massive cholera outbreak in 2012, followed by an Ebola outbreak 2014–2016, and the flooding and mudslides occurring in August 2017 have repeatedly tested the responsiveness and resilience of Sierra Leone’s health system. Since the end of the civil war in the early 2000s, Sierra Leone has made investments and commitments to health: sustaining a high level of government expenditure on health (10.84% of total government expenditure) compared to neighbouring countries [47], and receiving high levels of external investment. Despite these efforts, population health indicators such as maternal mortality and under-5 mortality continue to stagnate and are among the worst in the world [36, 48]. Given the wake of recent health system shocks [9, 25], an assessment of health sector fragmentation in Sierra Leone, and a critical look at the role of donor behaviour could be helpful for both better understanding current challenges and improving future responses. For the purpose of this article, health sector fragmentation is defined as: the coexistence of facilities or programmes that lack integration into the health network; or services at different levels of care that are not coordinated among themselves [50]. Fragmentation also refers to the existence of several separate funding mechanisms, and a wide range of health-care providers paid from various funding pools [16].

## Methods

The three examples described in this paper were purposefully selected with the goal of capturing manifestations and causes of health sector fragmentation spanning policies, programs, governance, and partnerships. Diversity between examples was prioritized in the selection process with the aim of presenting a snapshot of three distinct and varied illustrations of health sector fragmentation in Sierra Leone. Based on experiences of working in health systems strengthening in Sierra Leone, the authors collaboratively developed a short-list of possible suitable examples to profile. From the proposed examples, three were selected based on the following criteria (1) uniqueness of each example relative to one another (as described above) and (2) availability of literature to provide context and evidence for each example. Based on this, the following three examples were selected: 1) the community health worker (CHW) programme, 2) the national medical supply chain and 3) service level agreements.

A review of published and grey literature informed the elaboration, description, and analysis of these three examples. This review included searches conducted on PubMed using keywords “health sector fragmentation”, “Sierra Leone”, “external development assistance”, “health systems”, and “vertical funding”. A document review of Government of Sierra Leone (GoSL) and Ministry of Health and Sanitation (MoHS) policy and planning documents and reports was also included in this review. Of the documents reviewed and included in analysis, one third were academic journal articles, one third GoSL and MoHS documents, and another third other grey literature including reports from NGOs, research organizations, and multilateral agencies.

Authors’ experiences as participants in the health system processes, structures, and partnerships in Sierra Leone have also informed and influenced the analysis presented in this paper. The composition of authors includes a range of ‘outsider’ to ‘insider’ positionalities [41]; some authors bring years of experience working in health policy and planning environment in Sierra Leone and others a relatively ‘outsider’ perspective, with only short-term experience working in this context. All authors are non-Sierra Leonean nationals. The authors collaboratively developed the approach, analysis, and findings outlined in this paper. The authors’ positionality contributes a depth of contextual understanding while also biasing the analyses and findings according to our experiences, perspectives, and positions as participants in the dynamics discussed in this paper.

## Findings

### Factors contributing to health sector fragmentation in Sierra Leone

Health sector fragmentation is shaped (and sometimes exacerbated) by historical events and responses. The impact of international lending conditionalities and structural adjustment programmes on health systems and outcomes has been discussed extensively [3, 13, 15, 31, 37]. Health systems in several sub-Saharan countries haven’t recovered from the impact of structural adjustments and other economic reforms imposed by international agencies [3]. Many countries have a health sector under their Ministry of Health, as well as other parallel systems managed by donors and NGOs [3]. Sierra Leone has been a recipient of International Monetary Fund (IMF) support for over 20 years and the Government of Sierra Leone (GoSL) has reported being unable to meet social spending floors in areas including health due to lending conditionalities [13, 37]. The deprioritization of government spending on social sectors in response to these conditionalities has widened the gap between health sector needs and available domestic resources and paved the way for a high dependence on external funding in the sector [3, 13].

After the hostilities of a decade-long civil war ending in the early 2000s, in response to the weakened capacity within different line ministries, international staff were installed into key government systems to carry out core functions and ensure delivery of essential services [28]. This contributed to creation of parallel implementation structures, many of which still exist and continue to be recreated, such as multiple parallel sub-sector medical supply chains and health information systems [22, 28, 34]. Shortly after the civil war, between 2001 and 2006, Sierra Leone was the largest per capita recipient of foreign aid in the world [28]. With the recent Ebola epidemic, the country continues to receive assistance channeled through a variety of donors. Development assistance has been a substantial part of Sierra Leone’s economy, constituting over 9 % of the country’s Gross National Income (GNI) in 2013, and doubling during the height of the Ebola response [27].

The Ebola epidemic and its aftermath brought an influx of resources, activities, and attention to Sierra Leone’s health sector including an expansion of various actors and partners [22, 33, 49]. Given this attention, numerous critical documents, plans, strategies, and programmes have been developed and launched, with several more in the making. Since 2015, at least 20 health-specific strategic plans and policies have been launched [22]. These rapidly produced documents have been developed to varying standards of integration and overlap; and so, as the health sector has become more diverse, it has also become increasingly fragmented. MoHS resources and capacity do not match the scale or scope of these planned activities which challenges the feasibility of implementation [22]. The Ebola-response and subsequent influx of resources and policy and plan development also expanded the presence and involvement of partners in health and development sectors. As Sierra Leone approaches more than 2 years since the end of the Ebola outbreak, donor and partner support is diminishing - in some cases without adequate exit strategies. This context challenges the health sector coherence, coordination among stakeholders, and efficiency in allocation of limited resources.

### Examples of health sector fragmentation in Sierra Leone

In this section, we describe the CHW programme, national medical supply chain, and service level agreements (SLAs). These three examples illustrate both the impacts of, and factors contributing to health sector fragmentation in Sierra Leone. These examples also illustrate how fragmentation in the health sector exists at the level of policy and planning and extends into MoHS structures and across donor and partner relations.

#### Fragmentation in health sector policy and planning

Following the Ebola crisis, there has been an unprecedented amount of rapid policy and plan development in recent years [22]. The organization of these guiding documents at the level of MoHS policy and planning contributes to distortion, dilution, and diversion of health sector funding into numerous avenues of often overlapping or inadequately integrated vertical programmes [22]. We describe the national CHW programme as an example of a programme with integration and sustainability challenges due to policy and planning fragmentation, vertical funding and implementation, and donor and partner dependence.

##### Example 1: community health worker programme

The CHW programme was officially introduced by UNICEF, USAID, JSI, and the World Bank (among others) with the MoHS in 2012 to strengthen primary health care delivery at the community level [19]. The emergence of this cadre of health worker, which first appeared as a series of NGO-led vertical programmes [10, 18], was partly to overcome the reality that the previously established Maternal Child Health (MCH) Aides, who were intended to fulfill the role of CHWs, were not able to perform this role. Due to critical health workforce shortages and rural-urban disparities in Human Resources for Health (HRH), MCH Aides became attached to primary care facilities instead of operating in communities as initially intended. The estimated 15,000 CHWs are not part of the National Civil Service [18] and have not been integrated into the formal health sector [26], meaning they are not on payroll and do not receive any formal benefits from the MoHS.

Although officially overseen by the MoHS, the CHW programme is heavily dependent on donors and partners for technical, operational, and financial support. All programme costs are currently covered by donors. The CHW Policy 2016–2021 [19] indicates that the MoHS will take over financial and operational responsibilities for the programme, but in the short-term will continue to rely on donors and partners for financial, technical, and implementation support [19]. Within the MoHS, the National CHW Programme is overseen by a newly-established and donor-supported CHW Hub which falls under the Directorate of Primary Health Care - a separate entity from the Directorate of Human Resources for Health [2, 19]. In addition to the separation of CHWs from Human Resources for Health (HRH) in MoHS governance structures, CHWs are also addressed by a separate policy [19] as opposed to the recent HRH Policy [21] and HRH Strategy. This is one example of the expansion and diversification of MoHS structures, policies, and plans that has evolved in response to vertical donor funding.

#### Fragmentation in key health system structures

As reported by other global health initiatives, key barriers to improving service delivery include weak drug and medical supply systems [38, 42]. As seen in Sierra Leone, donors responses to this challenge often include establishing parallel supply chains to quickly meet the needs of their programme (Windisch, 2011; [6, 42]). Sierra Leone’s national medical supply chain has been profiled as an example of fragmentation in key health system structures.

##### Example 2: national medical supply chain

Sierra Leone’s national health sector supply chain management body, the National Medical Supplies Agency (NMSA) (formerly the National Pharmaceutical Procurement Unit (NPPU)), has recently undergone reform [18, 23]. DFID, World Bank, USAID, the Global Fund, and UNICEF supported the establishment of the newly formed NMSA [23]. One of the primary roles of the NMSA is to manage the distribution of Free Healthcare Initiative (FHCI) commodities; currently this is largely donor-funded and procurement and distribution are outsourced to UNICEF and Delivering Procurement Services for Aid (DPSA), respectively [18, 23]. Several additional parallel medical supply chains exist including those for specific programmatic areas HIV, TB, malaria, nutrition, and NTDs [18], which neither the NMSA, nor its predecessor NPPU, manage. The long-term mandate for the NMSA is to take over management of all partner-managed medical procurement and supply services. This was also intended to be within the mandate of the NPPU, however the technical and financial capacity of the NPPU was inadequate to fulfill this role [23]. Strengthening of the core national medical supply system is needed for the NMSA to grow into its mandate of managing all health sector supply systems including for non-FHCI MoHS, partner-operated, and donor-funded supply chains. This requires the establishment of the NMSA as a credible and accountable institution and, importantly, partners’ and donors’ willingness to integrate parallel supply chains and invest resources and capacity building in the central NMSA-managed system [23]. This would also be in alignment with the UHC 2030 IHP+ recommendation for development partners to “harmonize and align with national procurement and supply systems” as one of seven behaviours for effective development cooperation [3, 39]. Targeted efforts to integrate medical supply chains into existing structures in Sierra Leone would reduce duplication and streamline the medical supply chain to contribute to sustained overall systems efficiency and strengthening [30, 38].

#### Fragmentation in governance of partner engagement

A common theme emerging in the two examples discussed above are the challenges of managing, integrating, and governing partner engagement and health sector activities. Resulting from the Ebola recovery context, the GoSL has taken steps toward strengthening governance and oversight of these activities by introducing Service Level Agreements.

##### Example 3: service level agreements

In 2015, the GoSL launched Service Level Agreements (SLAs), agreements formed between partners and the GoSL, as a mechanism to enhance coordination and accountability of development partner activities [24]. SLAs have the potential to be a useful tool for health sector planning, alignment of activities with MoHS’ strategic priorities and plans, and reduced fragmentation. Although the GoSL introduced SLAs to streamline activities and improve coordination, they also represent another example of how health sector fragmentation has impeded the success of initiatives. In practice, SLAs have remained largely at the central level with inconsistent distribution to District Health Management Teams (DHMTs); thus, limiting the capacity of DHMTs to promote coordination and accountability among partners. An additional challenge is that SLAs are at times treated as a formality without adequate evaluation, follow up, or regulation [24]. Overlapping roles and responsibilities, communication barriers, and inconsistent devolution of power to DHMTs undermines the intent of the SLA approach and contributes to health sector inefficiencies.

DHMTs represent the MoHS in the districts and oversee planning, implementation, coordination, monitoring and evaluation of district health services and all primary health care [22]. While the responsibility for delivering basic services rests with the district level leadership (DHMTs and local councils), this responsibility has not been matched by a devolution of resources and decision-making power [6, 22]. Limitations to the autonomy of district level leadership challenge their capacity to be accountable for district-level health activities when they do not control the levers that would allow them to influence health management and outcomes [6, 22]. The current approach, involving a mix of central and district actors, has contributed to communication challenges and overlapping roles and responsibilities. Development partner practices have also played a role in the disempowerment of district level governance, contributing to fragmentation and inefficiencies in district activities [6, 7]. Duplication of activities, misalignment with domestic priorities, and limited health system integration of partner activities poses a challenge for health sector harmonization and coordination [6, 7].

## Discussion

The CHW programme, national medical supply chain, and SLA examples highlight fragmentation within health sector policy and planning processes, key health system structures, and governance mechanisms for partner engagement. Fragmentation at the level of policy and planning can be seen in the CHW programme and the parallel supply chains for disease-specific programme areas. The impact of the overlapping roles of local governing bodies and the incomplete decentralization of resources and authority for DHMTs to exercise the full extent of their roles impedes monitoring and accountability of district level health partnerships and undermines national structures and systems, as seen in the SLA example. Challenges in accountability and governance of partnerships and donor engagement result in inefficient allocation of scarce resources into vertical programmes that are often misaligned with national and community priorities. Although the GoSL has undertaken a number of activities to improve coordination and harmonization, several initiatives still operate as vertical programmes and lack integration into current systems and structures. Fragmentation of the international aid system with multiple donors and implementing partners remains an immense challenge in Sierra Leone.

In a review of the constraints to improving service delivery reported by several major global health initiatives, a key barrier is weak drug and medical supply systems [38, 42]. Often implementing partners’ response to this challenge is to establish supply systems parallel to the national supply chain to adequately and rapidly meet the needs of their programme versus investing in strengthening the existing national procurement and supply management system (Windisch, 2011; [30, 38]). The result is the emergence of several parallel sub-sector supply chains within the broader health system, as seen in Sierra Leone [30, 38]. This fragmentation dilutes resources and capacity, duplicates activities and creates administrative inefficiencies [38, 42].

Reliance on external assistance is a theme across each of the examples profiled above and perpetuates fragmentation and causes inefficiencies within the health sector. Dependence on external development partners has been shown to jeopardize the sustainability of existing progress, undermine national and community ownership, and distort the national agenda in other contexts including Cambodia, Pakistan, and other countries in sub-Saharan Africa [1, 14, 29]. It has been noted in other sub-Saharan African contexts that systems weaknesses, gaps in local capacity, and perceived lack of transparency have been found to contribute to the decision for external actors to implement parallel structures and vertical programmes that introduce health sector fragmentation [1, 4, 29, 32, 38, 42]. Efficient operation of external development assistance and reduced health sector fragmentation can be supported by harmonization of priorities and activities through policy and planning, commitment of external actors to strengthening national systems and structures, and greater accountability and trust in partnerships [1, 3].

### Way forward

#### Harmonization through policy and planning

A key opportunity for harmonization and provision of strategic direction to health sector activities is in national policy and planning. The MoHS recently completed the National Health Sector Strategic Plan (NHSSP 2017–2021) with the goal of providing a coherent vision and overarching framework to guide the direction and organization of health sector activities and actors [22]. This vision outlines national health priorities, objectives, and activities and provides a framework for donor investment and partner engagement to be aligned. The NHSSP provides an opportunity to highlight and harmonize overlapping or duplicated efforts; identify gaps in services, programming, and resources; and orient short-term activities and programmes within a longer-term plan for strengthening of health systems and structures. For example, as shown by international experience, until CHW training and remuneration are absorbed by the MoHS and more broadly integrated into the existing health system, the sustainability of the programme and the corresponding community services are at risk [8, 17, 40]. In a landscape of unpredictable and varied durations of external engagement and investment, continuity of health sector activities towards this longer-term vision rests with domestic actors (Bertone, Wurie, Samai & Witter, 2015). It has been noted that internal divisions between the central MoHS, and subnational levels exist further impeding sustainable progress from domestic actors [7].

In Sierra Leone, efforts to provide strategic direction for health sector inputs could be strengthened through establishment of a formal health financing strategy (this has not yet been developed [20], followed by a mapping exercise of health sector resources to inform an updated NHSSP costing framework. Effectiveness of the NHSSP as a tool for harmonization will also depend on monitoring of implementation, feedback and communication across levels of health sector actors, and the commitment, adaptability, and responsiveness of health sector leaders, managers, and partners to align policies with on-the-ground realities of implementation [3].

#### Commitment of external actors to strengthening of national systems and structures

Effective harmonization towards greater efficiency of health sector activities is a collaborative effort that depends on partner and donor responses to national priority setting and policy development and commitment to supporting and strengthening local systems and structures [32]. Global literature suggests that vertical, duplicated, and fragmented structures and programmes (such as the parallel supply chains and CHW programme described in the examples above) often emerge in response to system weaknesses, gaps in local capacity, and perceived unreliability or inefficiency of local systems and structures [4, 32, 38]. Moving away from the cycle of external intervention of this nature depends on investment in strengthening local systems, structures, and capacity [1, 3, 14]. Partners have an important role to contribute to this through filtering funding and operations through the existing health system structures for programme and service delivery [3]. Working with local human resources is an opportunity to support mutual learning and capacity building as long as partners are mindful to not drain limited national human resources from the public sector [1, 4].

#### Strengthening governance, mutual accountability, and partnerships

A final requisite for harmonization of a fragmented health sector is accountability across health sector relationships and partnerships [3]. The creation of parallel structures, programmes, and funding mechanisms are often precipitated by mistrust and poor communication among health sector actors. Strengthening of collaborative relationships can be facilitated through formalization of processes and pathways for communication and information sharing throughout MoHS governance structures and across health sector partnerships. As noted in Cambodia and Pakistan, creating joint policy platforms led by the MoHS are one possible way to enhance coordination of health sector stakeholders, broaden the collective policy-influence and power that is often held by health development partners, and strengthen mutual accountability [14]. Building on these relationships and communication pathways, as key elements of health system ‘software’ [3, 11], creates a foundation for accountability mechanisms to be strengthened. Small steps can be taken to strengthen accountability and harmonization of donor-funded and partner-implemented activities in Sierra Leone. This includes better leveraging of the SLA approach to coordinate, align, and monitor partner activities through more rigorous follow-up and evaluation of SLAs and stronger utilization of accountability mechanisms holding partners to their planned direction and activities. SLAs are a tool to ensure appropriate sustainability measures are in place, and responsible exit strategies. This will not be possible without the distribution of adequate resources, communication, and authority to district level leadership.

## Conclusion

As the health sector in Sierra Leone has become more diverse, it has also become further fragmented. This fragmentation continues to challenge the efficiency and coherence of health sector activities and emergency response capacities and impedes sustained health system strengthening [12]. The CHW programme, parallel medical supply chains, and SLAs illustrate this fragmentation in policy and planning, service delivery and governance structures, and across partner relationships. As seen in other countries, challenges within the health sector include the potentially irreversible dynamics of external assistance favouring disease-specific interventions and creation of parallel structures and programmes while neglecting a long-term vision for health systems strengthening [1, 14, 42]. Health sector fragmentation and distortion of priorities related to external development assistance continue to limit development progress, undermine the effectiveness of health systems strengthening efforts, and contribute to the inefficient use of scarce health sector resources.

Moving forward from these development patterns towards a more integrated and harmonized health system is a shared responsibility that will require commitment on the side of GoSL and MoHS leadership, external partners, and donors. Leveraging of health sector resources and partnerships in the short and medium term will support a transition away from the trend of external reliance towards strengthened systems and greater domestic independence, influence, and ownership of health sector priorities and activities. Efficient use of health sector inputs, particularly external assistance, can be supported by harmonization and assertion of national health priorities and objectives through policy and planning, collaborative partnerships to strengthen local systems and structures, and governance of health sector partnerships.

As emphasized in the *Lancet Commission on the future of health in sub-Saharan Africa*, comprehensive system-wide approaches, “home-bred solutions”, community and national ownership of health agendas, commitment to accountability, and alignment of domestic and external resources with the national health strategy are all critical ingredients for sustained strengthening of health systems, reduction of fragmentation and inefficiencies, and ultimately improvement of population health outcomes [1, 3]. These key messages and recommendations resonate with the findings of this case study of health sector fragmentation in Sierra Leone.

## Abbreviations

CHW: Community health worker
DFID: Department for international development
DHMT: District health management team
DPSA: Delivering procurement services for Aid
FHCI: Free healthcare initiative
GNI: Gross national income
GoSL: Government of Sierra Leone
HRH: Human resources for health
IHP: International health partnership
IMF: International monetary fund
JSI: John Snow Inc.
MCH: Maternal child health
MoHS: Ministry of health and sanitation
NHSSP: National health sector strategic plan
NMSA: National medical supplies agency
NPPU: National pharmaceutical procurement unit
NTD: Neglected tropical diseases
OECD: Organization for economic cooperation and development
SLA: Service level agreement
SSL: Statistics Sierra Leone
UHC: Universal health coverage
USAID: United States Agency for International Development
